# Effects of azithromycin in young adults with cystic fibrosis: a protocol for emulating a published randomised controlled trial using registry data

**DOI:** 10.1136/bmjopen-2024-091357

**Published:** 2025-03-25

**Authors:** Emily Granger, Jonathan Todd, Susan Christine Charman, Elizabeth Cromwell, Gwyneth Davies, Freddy Frost, Alex Gifford, Bin Huang, Nicole Mayer Hamblett, Lutz Naehrlich, Josh Ostrenga, Sanja Stanojevic, Rhonda Szczesniak, Ruth Keogh, CF Trial Emulation Network (CF-TEN), Ruth Keogh

**Affiliations:** 1 Department of Medical Statistics, London School of Hygiene & Tropical Medicine, London, UK; 2 Cystic Fibrosis Foundation, Bethesda, Maryland, USA; 3 Cystic Fibrosis Trust, London, UK; 4 Population, Policy and Practice Research and Teaching Department, UCL GOS Institute of Child Health, London, UK; 5 Liverpool Centre for Cardiovascular Science, University of Liverpool, Liverpool, UK; 6 University Hospitals Cleveland Medical Center, Cleveland, Ohio, USA; 7 University Hospitals Rainbow Babies & Children’s Hospital, Cleveland, Ohio, USA; 8 Division of Biostatistics and Epidemiology, Cincinnati Children’s Hospital Medical Center, Cincinnati, Ohio, USA; 9 Department of Pediatrics, University of Cincinnati, Cincinnati, Ohio, USA; 10 Seattle Children’s Research Institute, Seattle, Washington, USA; 11 Department of Pediatrics, Justus-Liebig-Universitat, Giessen, Germany; 12 Department of Community Health and Epidemiology, Dalhousie University, Halifax, Nova Scotia, Canada

**Keywords:** Cystic fibrosis, REGISTRIES, Observational Study, STATISTICS & RESEARCH METHODS

## Abstract

**Introduction:**

Target trial emulation is a framework for evaluating the effects of treatments using observational data. The trial emulation approach involves specifying key elements of a protocol for a target trial (a randomised controlled trial designed to address the question of interest) and then describing how best to emulate the trial using observational data. Recent years have seen an uptake of target trial emulation in several disease areas, although there are limited examples in cystic fibrosis (CF). This protocol describes a study which aims to assess the applicability of target trial emulation in CF. We aim to emulate an existing trial in CF and assess to what extent the results from the trial can be replicated using registry data.

**Methods and analysis:**

The target trial is a published randomised controlled trial which found evidence for beneficial effects of azithromycin use on lung function in young adults with CF. Two emulated trials are planned: one using data from the UK CF Registry and one using data from the US CF Registry. The inclusion and exclusion criteria, treatment and outcome definitions, follow-up period, and estimand of interest are all designed to match the published trial as closely as possible. The analysis step of the trial emulations will use causal inference methods to control for confounding. Results obtained in the emulated trials using registry data will be compared with those from the target trial.

**Ethics and dissemination:**

Ethical approval has been granted by the London School of Hygiene and Tropical Medicine Ethics Committee (Ref: 29609). This study has also been approved by the UK CF Registry Research Committee and the North Star Review Board. The results of this study will be published in a peer-reviewed journal and presented at relevant scientific conferences.

**STRENGTHS AND LIMITATIONS OF THIS STUDY**

- We use data from the UK and US Cystic Fibrosis (CF) Registries. These are the two largest national CF registries, and the UK CF Registry is cited as an exemplar patient registry in the National Institute for Health and Care Excellence real-world evidence framework.
- We use the target trial emulation approach. This approach helps to clearly articulate the study design and to avoid certain biases. We provide an example of target trial emulation in a disease area where there are limited applications of this approach for estimating treatment effects.
- The CF registries do not contain data on treatment doses or adherence, which limits our ability to match the treatment strategies in the target trial precisely.
- The CF registries do not contain data for all secondary outcomes used in the target trial.

## Rationale and background

 Randomised controlled trials (RCTs) are the gold-standard approach for evaluating the effects of treatments. However, RCTs are costly, and sufficiently large trials are not always feasible, particularly in patient populations with a rare disease, such as cystic fibrosis (CF). When an RCT is not feasible, an alternative is to use observational data to ‘emulate’ a trial.^1^ The trial emulation approach involves specifying key elements of a protocol for a target trial (an RCT we would like to conduct, if it were feasible) and then describing how best to emulate the target trial using the observational data at hand. This approach combines the study design principles of RCTs with an analysis appropriate for observational data.

Recent years have seen an uptake of target trial emulation in several disease areas.^2^,^7^ There is also rising interest in emulating existing RCTs in an attempt to replicate the results from the existing RCTs using observational data. The RCT DUPLICATE initiative recently published the results of 32 trial emulations using insurance claims data to replicate existing trials.^8^ They found that emulated trials based on insurance claims data can obtain similar results to the original RCTs. Matthews *et al*^9^ used Swedish registry data to emulate the Thrombus Aspiration in ST-Elevation Myocardial Infarction in Scandinavia randomised trial.^10^ Admon *et al*^11^ used target trial emulation to predict results of the Preventing Hypoxaemia with Manual Ventilation during Endotracheal Intubation Trial^12^ before they were published.

Despite the widespread use of target trial emulation across other areas of medicine, there are limited applications within the CF literature^13 14^; thus, its applicability to CF remains unclear. We aim to assess the applicability of target trial emulation in CF using data from the UK and US CF patient registries by emulating a published RCT within CF and assessing the extent to which the RCT findings could be replicated. Here, we set out the trial emulation protocol, including the statistical analysis plan. We follow the reporting guidelines recommended in the HARmonised Protocol Template to Enhance Reproducibility.^15^

## Research question and objectives

The primary objective is to emulate a published RCT of the effects of azithromycin in young adults with CF by Clement *et al*^16^ using observational data from two patient registries and to assess the extent to which the RCT results can be replicated. The RCT of Clement *et al*^16^ provides the target trial that this study aims to emulate. This trial was selected as we anticipate that it will be possible to replicate using the UK and US CF Registry data, based on our knowledge of the treatment and outcome data recorded. Table 1 summarises the research question addressed in the target trial.

**Table 1:** Description of the primary research question addressed in the target trial (Clement *et al*^16^)

Objective:	Investigate whether long-term use of azithromycin is associated with respiratory benefits in young people with CF
Population:	CF patients aged older than 6 years and forced expiratory volume in 1 s (FEV_1_%) of 40% or more.
Exposure:	Oral azithromycin
Comparator:	Placebo pills
Primary outcome:	Change in FEV_1_%
Time:	12 months
Setting:	Patients recruited from 18 CF accredited care centres in France
Main measure of effect:	Difference in change in FEV_1_% from baseline between treatment groups

CFcystic fibrosis

## Data sources

### UK CF registry

The UK CF Registry was established in 1995 and is a national database sponsored and managed by the CF Trust, with UK National Health Service research ethics approval. It records longitudinal data on approximately 99% of people with CF in the UK.^17 18^

Data are collected on time-invariant variables, such as sex at birth, cystic fibrosis transmembrane conductance regulator (*CFTR*) genotype, date of birth, diagnosis data and longitudinal variables that change over time. Longitudinal data are collected at approximately annual review clinic visits on over 250 variables covering several domains. These include clinical measurements taken on the day, and other variables covering the previous 12 months period such as: hospital admissions, treatments prescribed, culture and microbiology, health complications, nutrition, physiotherapy, smoking and outcomes (death and transplants). In 2016, the UK CF Registry started collecting treatment prescription start and stop dates.

### US CF registry

The US CF Registry began collecting data on people with CF in the USA in 1986 and is managed by the CF Foundation. It contains longitudinal information on approximately 80% of people with CF in the USA.^19^,^21^

Data are collected on demographic characteristics and on longitudinal variables that change over time. Data collection takes place at ‘encounter visits’ at CF care centres, with data also being abstracted annually. This study will use the encounter visit data which includes relevant information regarding hospitalisations, clinical measurements, medication usage, culture and microbiology, health complications. The encounter visits include routine clinical visits and visits in a hospital or the individual’s home. The non-routine hospital and home intravenous visits may be due to an individual experiencing worsening of their respiratory symptoms, and therefore, their lung function may be unstable at this time. These are referred to as ‘unstable’ visits, whereas routine clinical visits are referred to as ‘stable’ visits. The analyses in this study will use data from the stable visits only.

## Research methods

### Study design

We will conduct two studies nested within existing longitudinal data sets (one using UK CF Registry data, one using US CF Registry data), designed using the target trial emulation framework. Table 2 summarises the key components of the protocols for the target trial and the emulated trials.

**Table 2:** Summary of key components of the protocol for the target trial and emulated trials

Protocol component	Target trial based on Clement *et al*^**16**^	Emulation of the target trial using UK CF registry data	Emulation of the target trial using US CF registry data
Eligibility criteria	Include: French individuals diagnosed with CF (sweat chloride >60 mmol/L or a genotype known to cause the disease), aged 6–21 years, with the ability to perform pulmonary function tests with FEV_1_%>40 and the ability to swallow tablets.Individuals were excluded if they had the following: Allergy to macrolide antibiotics Long-term (>3 months) with macrolides during the 12-month period before study entry Liver disease with liver function tests >2 times the laboratory upper limit History of portal hypertension Kidney disease with serum creatinine >150 µmol/L and/or creatinine clearance <50 mL/min Use of any of the following in the 3 months before study entry: DNase, inhaled tobramycin, inhaled steroids	Individuals will be considered for inclusion if they have a clinically confirmed diagnosis of CF (ie, are present in the UK CF Registry) and have an observation date within the recruitment periods defined in section 4.3.1, aged between 6 and 21 years, and obtained FEV_1_%>40 on their pulmonary function test (taken on the day of the annual review). It is assumed that all individuals have the ability to swallow tablets.Exclusion criteria are as follows: Intolerance to macrolide antibiotics recorded at any time during study period. Prescription of chronic oral or prophylactic oral macrolides (including azithromycin) recorded at time 0. Acute liver failure with >3×the upper laboratory limit, INR>2, or not responsive to vitamin K at time 0 Recorded cirrhosis with portal hypertension at time 0. Serum creatinine levels >150 µmol/L at time 0. Prescription of DNase, inhaled tobramycin or inhaled corticosteroids recorded at time 0. No follow-up visit for time 1.	Individuals will be considered for inclusion if they have a clinically confirmed diagnosis of CF (ie, are present in the US CF Registry) and have an observation or encounter date within the time periods defined in section 4.3.1, aged between 6 and 21 years, and obtained FEV_1_%>40 on their pulmonary function test (taken on the day of the encounter visit). It is assumed that all individuals have the ability to swallow tablets.Exclusion criteria are as follows: As in the UK Emulated Trial As in the UK Emulated Trial Non--related liver disease recorded at time 0. Laboratory results from liver tests are not available in the US registry. Recorded cirrhosis at time 0. Portal hypertension is not available in the US registry. As in the UK Emulated Trial As in the UK Emulated Trial As in the UK Emulated Trial
Treatment strategies	The active intervention was azithromycin supplied as 250 mg tablets and the comparator was placebo pills.Individuals weighing less than 40 kg took one tablet 3 days per week, and individuals weighing more than 40 kg took two tablets 3 days per week.	The active intervention is prescription of oral azithromycin and the comparator is no prescription of oral azithromycin.Further details are provided in the section titled "Treatment strategies".	
Assignment procedures	Individuals were randomised to treatment strategy. Randomisation was stratified according to centre and *Pseudomonas aeruginosa* infection status. The patients and all study investigators remained blinded to the treatment assignment until study completion.	In the emulated trials, individuals are not randomly assigned to the treatment strategy. This is accounted for in the analysis.	
Follow-up period	12 months	As in the target trial	
Outcome	Primary outcome: mean change in FEV_1_% between month 0 and month 12.Secondary outcomes included: evaluation of the number of pulmonary exacerbations, the use of antibiotics, modifications of microbiological analysis of sputum or throat cultures, changes in FVC, nutritional status with measurement of body mass index (BMI) and quality of life.	Primary outcome: absolute FEV_1_% at the end of follow-up.Secondary outcomes include: prescription of intravenous antibiotics, FVC, BMI z-score.Further details are provided in the section titled "Outcomes".	
Causal contrasts of interest	Intention to treat	Per-protocol	
Analysis plan	For continuous outcomes, mean differences between treatment groups were estimated using mixed models; for binary outcomes, logistic regression was used; for count outcomes, Poisson regression was used.	A direct acyclic graph is used to inform which variables need to be controlled for (see sectiontitled "covariates").Confounding by measured variables will be accounted for using inverse-probability-of-treatment weighting.Further details are provided in the section titled "Data analysis".	

CFcystic fibrosisFEV_1_forced expiratory volume in 1 sFVCforced vital capacity

### Setting

#### Time periods

The target trial was conducted from 2001 to 2003, with results published in 2006. Within the data collection period for the target trial, results from other azithromycin RCTs were published,^22^,^24^ after which there was uptake of this treatment in routine clinical practice. We plan to emulate the trial using data from three time periods of 3 years’ duration: 2003–2005, 2007–2009, 2016–2018. Period 1 is close to the timing of the target trial while allowing time for the treatment to have come into use. Periods 2 and 3 were chosen based on features of the data, and ending the time frame in 2018 means that we only use data from the time before CFTR modulators became widespread in clinical practice.

Table 3 provides further details and justification about the three time periods. For each 3-year time period, the first 2 years are used as the ‘recruitment period’, defined as the period during which individuals are considered for inclusion in the emulated trial. The target trial recruited participants over 2 years. Individuals are included in the emulated trial data if they meet the inclusion and exclusion criteria in at least one of their visits during the recruitment period.

**Table 3:** Description of the time periods considered in the UK and US registry data

Time period	Justification for time period	UK data	US data
2003–2005	Closest time period to the target trial, allowing a couple of years for use of azithromycin to uptake in clinical practice.This will only be conducted using the UK data as the US registry did not collect data on azithromycin use during this time.	✓	×
2007–2009	The US registry started collecting data on azithromycin in 2006 and a 1-year wash-out period is required to select individuals who were not taking azithromycin prior to study entry. Therefore, this time period is as close to the time period used in the target trial as is possible for the US registry.	✓	✓
2016–2018	The UK registry started collecting data on treatment prescription dates in 2016. Therefore, this time period is a more recent period that predates widespread use of CFTR modulators but also allows use of treatment date data.	✓	✓

CFTRcystic fibrosis transmembrane conductance regulator

#### Definition of time 0 and the index visit

Time 0 is defined as the time at which individuals meet the eligibility criteria and ‘enter’ the emulated trial, analogous to the time of randomisation in the target trial. Time 1 is 12 months postbaseline, and the outcome of interest is forced expiratory volume in 1 s (FEV_1_%) at time 1.

**Figure 1:**
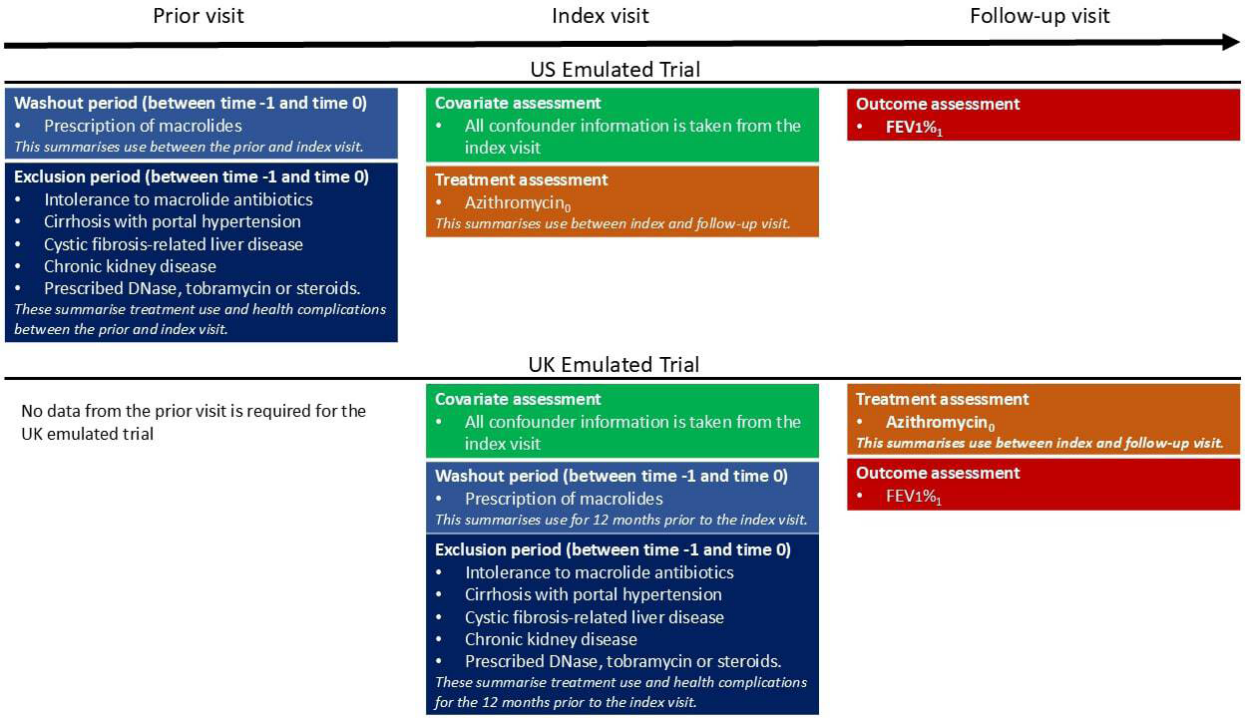
Study design diagram illustrating from which visits data are extracted for time 0 and time one in the UK and US Emulated Trials. Subscripts denote time. FEV_1_, forced expiratory volume in 1 s.

For the emulated trials conducted within period p (p=1,2,3), we define an ‘index visit’ for each individual who meets the eligibility criteria during the recruitment period, such that the date of the index visit is time 0. Follow-up visits take place approximately 12 months after the index visit, and an eligibility criterion is that individuals are required to have a follow-up visit. Index visits and follow-up visits are defined differently in the UK and US emulated trials, due to differences in data collection between the two registries. Moreover, the visit we use for treatment, outcome and covariate data at time 0 and time 1 differs between the UK and US emulated trials; figure 1 summarises these differences.

##### UK CF Registry Data

1. Using annual review data only

Within the period p, the index visit for a given individual is the first annual review visit at which they meet the eligibility criteria in the 2-year recruitment period. The follow-up visit is the date of the next annual review which is closest to 12 months after time 0, but falls within 9–15 months after time 0. The outcome is FEV_1_% measured on the day of the follow-up visit. Individuals are included in the treated group if they are recorded as being prescribed azithromycin at the follow-up visit because the information recorded on treatment use at the annual review refers to treatment use over the past year. Individuals not recorded as being prescribed azithromycin at the follow-up visit are included in the control group.

1. Using annual review data and prescription dates data

In the UK Registry, prescription dates data are available from 2016 onwards. Therefore, in period 3 (2016–2018), we can conduct a second analysis making use of the dates data for a more precise time 0. In the second analysis, the index and follow-up visits are defined as above for individuals in the control group. For treated individuals, the index visit is defined similarly; however, time 0 is defined as the first date post-index visit at which azithromycin is prescribed. The follow-up visit is defined as the next annual review visit which is closest to 12 months after time 0, but falls within 9–15 months after time 0.

##### US CF Registry Data

Within the period p, the index visit is defined as the first stable encounter visit at which an individual meets the eligibility criteria in the 2-year recruitment period. The follow-up visit is defined as the date of the stable encounter visit that is closest to 12 months after the index visit, but which falls within 9–15 months after the index visit. Individuals are included in the treatment group if they are recorded as being prescribed treatment at the index visit, as this is often assumed to be the treatment start date. Once an individual starts treatment, we assume they remain on treatment until the follow-up visit. Individuals are included in the control group if they are recorded as not being prescribed azithromycin at the index visit. Controls who are recorded as starting treatment at an encounter visit between the index visit and the follow-up visit are censored at the date of that encounter visit. For the US emulated trials, we also define the prior visit to be the most recent stable encounter visit prior to the index visit.

### Inclusion and exclusion criteria

Table 2 summarises the inclusion and exclusion criteria for the target and emulated trials. The data on liver function test results and serum creatinine levels (exclusion criteria 3 and 5) may have large amounts of missingness and therefore be unusable. An alternative is to use indicator variables for any recorded non-CF-related liver disease (for exclusion criteria 3) or chronic kidney disease (for exclusion criteria 5).

### Variables

#### Treatment strategies

The active and comparator treatment strategies used in the target trial are provided in table 2. We aim to match these strategies as closely as possible; however, the target trial specifies doses and frequency of treatment, and this information is not available in the UK or US CF registries.

For both the UK and US emulated trials, the active treatment is prescription of prophylactic oral or chronic oral azithromycin and the comparator is no prescription of prophylactic oral or chronic oral azithromycin.

#### Outcomes

Where possible, the emulated trials will replicate outcomes studied in the target trial; however, data are not available in the registries for all secondary outcomes. The primary outcome in the target trial, and both emulated trials, is absolute FEV_1_% at time 1. Secondary outcomes in the target trial include: number of pulmonary exacerbations, forced vital capacity (FVC), nutritional status with body mass index (BMI) z-score, the use of antibiotics, modifications of microbiological analysis of sputum or throat cultures and quality of life. Secondary outcomes in the emulated trials include:

- Prescription of intravenous antibiotics at time 1 (as a proxy for pulmonary exacerbations).
- Percent predicted FVC at time 1.
- BMI z-score at time 1.

For the emulated trials, FEV_1_% will be calculated using the Global Lung Initiative (GLI) 2012 equations^25^ and BMI z-scores will be calculated using the WHO reference distribution.^26^

#### Covariates

**Figure 2:**
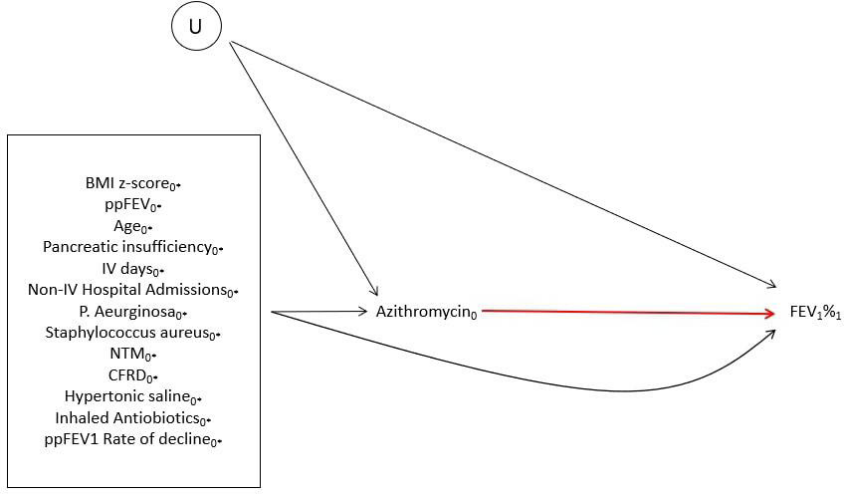
Directed acyclic graph depicting assumed confounding relationships for the association between azithromycin at time 0 (azithromycin0) and FEV1% at time 1 (FEV1%1). U represents any unmeasured confounders. Subscripts denote time; 0* indicates prebaseline. BMI, body mass index; CFRD, cystic fibrosis-related diabetes; FEV_1_, forced expiratory volume in 1 s; NTM, nontuberculous mycobacteria; ppFEV_1_, per cent predicted FEV1.

In the target trial, individuals were randomly allocated to the treatment or placebo strategy. In the emulated trials, there is no randomisation. The data on treatment use within the UK and US CF Registries reflects treatment decisions made based on clinical indication and on clinician and patient preference. Being prescribed azithromycin is, therefore, assumed to be informed by a number of factors, many of which are also associated with the outcomes of interest. The association between prescription of azithromycin and FEV_1_% (and secondary outcomes) is, therefore, believed to be confounded by the following factors: age, number of days on intravenous antibiotics (intravenous days), non-intravenous hospital admissions, presence of *Pseudomonas aeruginosa*, *Staphylococcus aureus* or Nontuberculous Mycobacteria, pancreatic insufficiency, CF-related diabetes (CFRD), use of hypertonic saline or inhaled antibiotics and rate of decline in FEV_1_%, BMI z-score and FEV_1_%. These are depicted in a directed acyclic graph in figure 2.

Age, FEV_1_% and BMI z-score are continuous. Rate of decline in FEV_1_% is calculated as the difference between the absolute FEV_1_% measured at the index visit and the prior visit. Data on treatment prescription, the presence of infections, CFRD diagnosis, pancreatic insufficiency and non-intravenous hospital admissions will be binary indicators. Indicators for pancreatic insufficiency and non-intravenous hospital admissions are created using existing variables in the data. Registry data provide dates for treatment with intravenous antibiotics (at home or hospital). These data will be used to create a variable indicating the number of days on intravenous antibiotics since the last annual review (including treatment administered at home and hospital). Intravenous days will then be treated as a categorical variable with four categories: 0, 1–14, 15–28, 28+.

### Data analysis

The following data analysis plan will be implemented in both the UK and US emulated trials.

#### Notation

Let A denote an indicator variable for treatment strategy (A=0 indicates no prescription of azithromycin and A=1 indicates prescription of azithromycin). Let $Y_{t}\left(0\right)$ denote the potential outcome under treatment A=0 at time t,t∈{0,1} where t=1 is 12 months after t=0. Similarly, let $Y_{t}\left(1\right)$ denote the potential outcome under treatment A=1 at time t. Finally, C denotes the confounding factors listed in the section titled "Covariates" and in figure 2. In the following sections, we describe the causal estimand of interest, the main analysis plan with a focus on the primary outcome, and the key differences in the analyis plan for the secondary outcomes.

#### Causal estimand of interest

The target trial reported the difference in mean changes (between month 0 and month 12) in FEV_1_% between treatment groups in the total population:

(1) $$ATE=E\{Y_{1}(1)-Y_{0}(1)\}-E\{Y_{1}(0)-Y_{0}(0)\}$$

where the expectations refer to the population of individuals meeting the criteria for the target trial. This is equivalent to the difference in means at the end of follow-up as the observed value of Y at time 0 is unaffected by treatment, that is, $Y_{0}\left(a\right)=Y_{0},i.e.$

(2) $$ATE=E\{Y_{1}(1)\}-E\{Y_{1}(0)\}$$

Our causal estimand is interpreted as the expected difference in FEV_1_% at month 12 if everyone had taken azithromycin for 12 months, compared with a scenario where no one took azithromycin for 12 months.

#### Main analysis

In the target trial, the authors investigated the change in FEV_1_% from baseline, with adjustment for baseline FEV_1_%. This is equivalent to a regression of the mean FEV_1_% at follow-up with adjustment for baseline FEV_1_%. We note that in the absence of imbalance between treatment groups in baseline FEV_1_%, the adjustment of baseline FEV_1_% is not required but may result in gains in efficiency. Both approaches result in estimates of the ATE as defined in equations(1)  (2).

In the emulated trials, we need to account for differences between treatment groups at baseline, including baseline FEV_1_%. We also require assumptions of positivity, no interference, consistency and conditional exchangeability (conditional on C). We use augmented inverse-probability-of-treatment weighting (AIPTW) to control for potential confounding by C. AIPTW involves defining models for the treatment and outcome. This approach was chosen as it is doubly robust, meaning that it gives consistent estimates of treatment effects if either the treatment model or outcome model is correctly specified.^27^ This represents an advantage compared with the alternative propensity score or outcome-regression based approaches which are singly robust.

The first step in implementing AIPTW is to estimate the propensity scores, that is, the probability of treatment conditional on baseline covariates:

(3) PS=P(A=1|C)

Propensity scores will be estimated using logistic regression, including C as linear terms.

In the second step, we specify an outcome model conditional on treatment and covariates:

(4) $$Y_{1}=\gamma_{0}+\gamma_{A}A+\gamma_{C}C+\varepsilon$$

The AIPTW estimator for

(5) $$\hat{E}[Y_{1}(a)]=\frac{1}{n}\sum_{i=1}^{n}\left\{\frac{I(A_{i}=a)Y_{1i}}{\hat{P}(A_{i}=a|C_{i})}-\frac{I(A_{i}=a_{i})-\hat{P}(A_{i}=a|C_{i})}{\hat{P}(A_{i}=a|C_{i})}\hat{E}(Y_{1i}|A_{i}=a,C_{i})\right\}$$

where $\hat{P}(A_{i}=a_{i}|C_{i})$ can be obtained using predictions from the propensity score model. The estimator in equation 5 can be used to estimate $E\left[Y_{1}\left(0\right)\right]$ and $E[Y_{1}(1)],$ and the difference between these two expectations is an estimate of the average treatment effect in the population. Standard errors can be obtained based on the efficient influence function.^28^

##### Additional analysis in the UK Emulated Trial making use of data on treatment prescription dates

A limitation of this approach is that it assumes individuals with A=1 at time 1 have been taking azithromycin for the past 12 months. Realistically, individuals may initiate treatment with azithromycin at any time between time 0 and time 1. For the most recent time period, we can conduct a second analysis using the UK data and making use of data on treatment prescription dates.

In this second analysis, AIPTW is used as above, but the outcome model used previously (equation 3) is modified to include a variable indicating time and an interaction term between time and treatment. We define a new time variable, $t^{*}$, which measures time in months. For treated individuals, $t^{*}=0$ on the first date they are prescribed azithromycin after the index visit. For control individuals, $t^{*}=0$ for the date of the index visit. We let $Y_{t^{*}=12}$ denote FEV_1_% measured on the day of the annual review after $t^{*}=0$ and closest in time to $t^{*}=12.$ Note that this accommodates the fact that annual review visits do not always take place exactly 12 months apart. The outcome model will then be defined as:

(6) $$Y_{t^{*}=12}=\delta_{0}+\delta_{A}A+\gamma_{c}C+\delta_{t^{*}}t^{*}+\delta_{At^{*}}At^{*}+\epsilon$$

After fitting this model, we set $t^{*}=12$ to obtain the relevant expected outcomes.

##### Diagnostics

The distribution of weights will be assessed using summary statistics and plots. Methods such as trimming or truncating will be considered to deal with extreme weights. Standardised mean differences will be used to compare the balance in the distribution of confounders between treatment and control groups in the original and weighted samples.

### Secondary outcomes

The secondary outcomes are prescription of intravenous antibiotics, per cent predicted FVC (FVC%) and BMI z-score. Analysis of the continuous outcomes (FVC% and BMI z-score) can be implemented as described above. Prescription of intravenous antibiotics will be treated as a time-to-event outcome (time to first prescription of intravenous antibiotics at home or in hospital) where censoring occurs at 365 days, or prior in the event of death or organ transplant. HRs will be estimated using Cox regression for the outcome models.

### Sensitivity analyses

#### Sensitivity to the no unmeasured confounders assumption

Our analysis relies on the assumption that there are no unmeasured confounders. Unfortunately, there may exist some factors that are associated with both treatment prescription and outcome, which are not captured in the registries (denoted by U in figure 2). Sensitivity to unmeasured confounders will be summarised using E-values.^29^

#### Allowing individuals to enter the emulated trials more than once

For the main analysis, individuals will be included in the emulated trial once. Individuals ‘enter’ the trial at time 0, which is defined as the earliest year within the recruitment period that they meet the inclusion and exclusion criteria. This approach restricts the analysis to using information from everyone at one time point only and may be inefficient. Alternatively, we can allow individuals to ‘enter’ the trial twice if they meet the inclusion and exclusion criteria in both years during the recruitment period. Standard errors will need to take into account that individuals are included multiple times.

### Missing data

The amount of missing data in each variable will be summarised in tables by treatment group. Where there are missing data in binary time-varying variables that are usually static for long time periods, we will use a simple imputation approach. For missing visits where the prior visit and subsequent visit are equal, we will assume the missing value is also equal and impute accordingly. This approach will be used for the following variables: pancreatic insufficiency, *P. aeruginosa*, *S. aureus*, NTM, CFRD, inhaled antibiotics, inhaled steroids, hypertonic saline and DNase. Missingness patterns in the remaining missing data will be explored. If there are missing outcomes that are missing at random conditional on C, then a complete case analysis is appropriate.^28^ If a complete case analysis is not appropriate, more complex missing data methods such as multiple imputation by chained equations^30^ may be considered.

### Comparison of results against the target trial

We will compare our results with those from the target trial with the aim of determining whether results from the emulated trials are compatible with the target trial. The following criteria will be considered, as were used in the RCT DUPLICATE Project:^8^

1. Do the estimated ATEs from the emulated trials replicate the direction and statistical significance of the estimated ATE in the target trial?
2. Do the estimated ATEs from the emulated trials lie within the 95% CIs for the ATE estimates reported in the target trial?
3. Is there evidence against the null hypothesis of no difference between the ATE estimates from the emulated trials and those from the target trial? To assess this, we calculate the standardised mean difference between the effect estimate obtained in the target trial and that obtained in the emulated trial. Evidence against the null hypothesis at the 5% level is indicated by a standardised mean difference greater than 1.96.

### Patient and public involvement

Patients and the public were not involved in this research study. There are no plans for patient and public involvement.

## Limitations

There are a number of limitations to this trial emulation, which are sources of potential bias in our results, and which may explain any differences in findings between the target trial and the emulated trial. Here, we identify a number of sources of bias and/or potential reasons we may observe differences in the results, due to either limitations regarding data availability in the registries, differences in sample size or differences in the study populations.

### Data availability

The target trial specified a particular dose of azithromycin depending on an individual’s weight. The trial also reported high adherence, estimated at 95% for azithromycin and placebo. Neither the UK nor US Registry provides reliable data on treatment doses, and it is possible that individuals in the registry will take different doses to those given in the target trial (see table 2). There are also no data on adherence, and our emulated trial relies on data on treatment prescription, which may differ from actual treatment use.

Some of the exclusion criteria of the target trial cannot be replicated exactly in the emulated trial. For example, the target trial included a criterion based on liver function tests, with individuals excluded if they had liver disease with liver function tests more than twice the laboratory upper limit. In the UK Registry, the closest variable to this criterion is an indicator for acute liver failure with liver function tests greater than three times the laboratory upper limit. The US Registry has a similar variable, but data collection for this variable began in 2015, so it can only be used for the most recent time period.

The target trial calculated the outcome, FEV_1_%, using the Knudson equations^31^; we plan to use the GLI equations in the emulated trial,^25^ as these are now more commonly used. Previous research suggests that results will be minimally affected by choice of reference equations.^32^

The main analyses in the UK emulated trials will use data from consecutive annual review visits. We assume that the annual review visits are 12 months apart and that individuals in the treatment group were taking azithromycin for the 12 months in between visits. In practice, the annual review visits are not always exactly 1 year apart, and individuals may begin treatment with azithromycin at any time during the time between visits. We address this limitation to some extent in an additional analysis for the UK Registry data, in which we incorporate prescription date data.

Finally, our analysis relies on the assumption that all confounding of the treatment-outcome association is accounted for in the analysis. It is possible that there are some factors associated with both azithromycin prescription and the outcome that are not collected in the registry. We plan a sensitivity analysis to assess how sensitive our results are to unmeasured confounders.

### Sample size

The target trial included 82 individuals (40 in the treated group and 42 in the placebo group). The authors note in their discussion that it is possible the study was not adequately powered to detect significant differences in FEV_1_%.

We have not performed sample size calculations for the emulated trials, and there is some debate as to whether sample size calculations are needed in studies using observational data.^33^,^35^ We plan to use all the available data in the UK CF Registry or US CF Registry and expect much larger sample sizes than were used in the target trial.

### Differences in the study populations

Ideally, we would conduct the emulated trials using data from a similar time period as the target trial, to ensure homogeneity in the clinical settings. The target trial was conducted from 2001 to 2003, but azithromycin was not commonly used in clinical practice at this time. Therefore, for the emulated trials, we need to wait for the treatment to uptake in clinical practice, that is, after the earlier azithromycin trials were published in 2002.^22 23^ Additionally, in 2007, the UK CF Registry introduced a new web-based data collection system which improved data collection and data quality. Restricting the emulated trials to years prior to 2007 would, therefore, not make use of the years with higher data quality. On the other hand, using later years could result in differences in the clinical setting between the emulated and target trials. For this reason, we have suggested multiple time periods for the emulated trials and will compare results between time periods. Finally, due to the way the data are collected in the two registries, we require different definitions of time 0 for the UK and US emulated trials. The different definitions may lead to slightly different populations of interest and, therefore, the estimands between the UK and US emulated trials would be based on different populations. This could lead to different results between the emulated trials.

Since our goal is to investigate whether we can replicate the findings of the target trial using target trial emulation, the potential differences in study populations are limitations in the sense that they may lead to different results between the target and emulated trials. However, in other settings, these differences could be considered a strength of the target trial emulation approach. For example, using target trial emulation with observational data, we may be able to study more diverse or generalisable populations than is possible in an RCT, or study the effects of treatments in populations that are less represented in RCTs such as those with severe disease.

## Ethics and dissemination

This project will use anonymised data from the UK Cystic Fibrosis Registry, which has Research Ethics Approval (ref: 24/EE/0012) and from the US Cystic Fibrosis Registry. This protocol was reviewed by Advarra IRB and it was confirmed that no study-specific IRB approval was required to use the US Cystic Fibrosis Registry data. No additional data beyond that contained in the registries will be collected for the project. Ethical approval has been granted by the London School of Hygiene and Tropical Medicine Ethics Committee (Ref: 29609). The study has also been approved by the UK CF Registry Research Committee and the North Star Review Board.

This work is being undertaken by the CF Trial Emulation Network, a new multidisciplinary international collaborative network. We plan to publish the results of this study in a high-ranking peer-reviewed journal. Findings will also be presented at relevant scientific conferences such as the European Cystic Fibrosis Conference, the North American Cystic Fibrosis Conference and the International Society for Clinical Biostatistics.

This work will contribute to the evidence base for the target trial emulation approach in CF. If the trial emulations are a success, we could extend the research to study questions beyond the trial. For example, the longer-term effects of azithromycin, effects of azithromycin use on other outcomes such as risk of NTM infection or combination effects of multiple treatments. Such questions are often difficult to study in RCTs due to additional costs or lack of statistical power.
