# Supplementary material for: Effects of azithromycin in young adults with cystic fibrosis: a protocol for emulating a published randomised controlled trial using registry data
Source: BMJ Open. 2025 Mar 25;15(3):e091357. doi: 10.1136/bmjopen-2024-091357 (PMC11973775; doi:10.1136/bmjopen-2024-091357)
Supplement: Supplementary file 1 [file bmjopen-2024-091357.reviewer_comments.pdf]

PEER REVIEW HISTORY

BMJ Open publishes all reviews undertaken for accepted manuscripts. Reviewers are asked to complete a checklist review form and are provided with free text boxes to elaborate on their assessment. These free text comments are reproduced below.

ARTICLE DETAILS

Title (Provisional)

A protocol for emulating a published randomised controlled trial using registry data: effects of azithromycin in young adults with cystic fibrosis

Authors

Granger, Emily; Todd, Jonathan; Charman, Susan Christine; Cromwell, Elizabeth; Davies, Gwyneth; Frost, Freddy; Gifford, Alex; Huang, Bin; Hamblett, Nicole Mayer; Naehrlich, Lutz; Ostrenga, Josh; Stanojevic, Sanja; Szczesniak, Rhonda; Keogh, Ruth; Emulation Network, CF-Trial

---

VERSION 1 - REVIEW

**Reviewer**	**1**
**Name**	**Langton , Simon**
**Affiliation**	**Bristol Royal Hospital for Children**
**Date**	**08-Oct-2024**
**COI**	**None**

This paper outlines a protocol with a novel approach towards studying the effects of azithromycin in young adults. I really like the approach that is taken here where two very large CF registries that contain a vast about of data will be interrogate. The UK Registry contains a much more complete dataset than that of the US but despite this the US dataset is very large and includes data on over 80% of US patients with CF and so it seems a valid approach to use this large dataset in the manner outlined by the protocol. By publishing the protocol the authors are able clearly to state their intentions for the study. Conducting an RCT to address a similar question would be time consuming and expensive and so this alternative approach appears to have many merits. The study will have limitations which the authors acknowledge including the lack of randomisation or of blinding.

Despite these limitations I am happy to recommend this protocol for publication.

**Reviewer**	**2**
**Name**	**Landsittel, Douglas**

Affiliation **University at Buffalo**
Date **21-Dec-2024**
COI **None**

The submitted paper describes a protocol for emulating an RCT on effects of azithromycin in young adults with cystic fibrosis (CF). The proposed observational study utilizes data from both the UK and US CF registries. They propose to emulate a target trial by Clement, et al., that was published in 2006. This analysis appears to be part of, or related to, 1) the larger RCT DUPLICATE initiative, which recently published results of 32 target trial emulations, and 2) the CF Trial Emulation Network.

The general approach of target trial emulation is an important methodology that can better establish causal inferences specific to a given hypothesis and causal estimand. The paper sufficiently outlines the associated approach, including trial protocol details such as the inclusion and exclusion criteria, follow-up measurements, and definitions of time 0 and the treatment and outcome. The use of three different three-year intervals, while initially somewhat confusing to this reviewer, does seem necessary to account for the strong potential for temporal effects. They will also follow standard guidelines for reproducibility and appropriately describe use of propensity score-based methods for better estimation of the causal effect (i.e., as defined by the potential outcomes framework).

The two different observational registries, from the UK and US, seem to represent appropriate observational data sources, and are sufficiently well-described in the text. The associated limitations of those data, such as lack of some secondary outcomes, are also described in the approach. As part of that description, they also differentiate between stable and unstable visits and other aspects of the data collection that may affect bias in the more standard 'use all data' approach to observational research.

Overall, the protocol paper did not seem to have any significant limitations that will adversely affect quality of the eventual findings or should preclude the publication of this protocol. Some issues, however, should be better clarified in the manuscript, including the following:

Under the Article summary (and Strengths and limitations), they describe their example as one where there are "limited applications". It is not clear if this is saying there are limited observational data sets available for this goal or there are limited opportunities for RCTs, or something else.

Propensity score-based (PS) methods are just one approach for better approximating causal inferences with observational data, augmented inverse PS weighting is just one approach for better balancing confounders, and the logistic model is just one approach for the PS assignment model and for the outcomes model. A better justification should be included for each of these selections. It was also unclear to this reviewer why only confounders are used

in the AIPTW calculations (versus including both confounders and outcome predictors to improve precision).

One significant issue for interpretation was the apparent focus on any differences from the target RCT as weaknesses. In multiple places across the paper, they seem to focus on the goal of matching results from the published target trial. In fact, the limitations described in Section 8 seem to focus specifically on reasons for those differences. The Research question and objectives also list replication as a goal.

While this reviewer understands that achieving very similar results as the RCT is reassuring, finding different results may be a significant strength, e.g., through better representation of more diverse or generalizable study populations or through use of larger data sets that are better powered to detect significant relationships. While they do highlight some of these issues, they all seemed to be highlighted under the heading, or in the context of weaknesses not strengths. The conclusions or other sections of the manuscript should better reflect the benefits of target trial emulation beyond simply matching the original target RCT. Part of that benefit can be described through describing potential participant selection bias in the RCT versus the registries.

While randomization in an RCT does have clear advantages to giving an unbiased estimate of the causal effect, that estimate may be using a very biased population. While this reviewer understands that the approach in the established protocol cannot be modified now, it would be worth considering ad hoc analyses (where they are labeled as such) which evaluate whether the estimate of treatment effect varies across study populations that were less represented by the RCT. This may be difficult to accomplish however if the inclusion and exclusion criteria are driving those biases in the enrolled RCT population. At the very least, this point should be further described in the conclusions or elsewhere in the paper.

VERSION 1 - AUTHOR RESPONSE

Reviewer 1: Dr Simon Langton

This paper outlines a protocol with a novel approach towards studying the effects of azithromycin in young adults. I really like the approach that is taken here where two very large CF registries that contain a vast about of data will be interrogate. The UK Registry contains a much more complete dataset than that of the US but despite this the US dataset is very large and includes data on over 80% of US patients with CF and so it seems a valid approach to use this large dataset in the manner outlined by the protocol. By publishing the protocol the authors are able clearly to state their intentions for the study. Conducting an RCT to address a similar question would be time consuming and expensive and so this alternative approach appears to have many merits. The study will have limitations which the authors acknowledge including the lack of randomisation or of blinding.
Despite these limitations I am happy to recommend this protocol for publication.

Thank you for your comments on our protocol.

Reviewer 2: Prof. Douglas Landsittel

The submitted paper describes a protocol for emulating an RCT on effects of azithromycin in young adults with cystic fibrosis (CF). The proposed observational study utilizes data from both the UK and US CF registries. They propose to emulate a target trial by Clement, et al., that was published in 2006. This analysis appears to be part of, or related to, 1) the larger RCT DUPLICATE initiative, which recently published results of 32 target trial emulations, and 2) the CF Trial Emulation Network.

Thank you for your comments on our protocol. This work is part of the CF Trial Emulation Network, which we reflect in the author list, but we are not connected to the RCT DUPLICATE initiative. However, like in RCT DUPLICATE, as well as other studies in different disease areas, we are aiming to replicate findings of a published RCT. Additionally, we use the same performance metrics to those used in the RCT DUPLICATE project.

Overall, the protocol paper did not seem to have any significant limitations that will adversely affect quality of the eventual findings or should preclude the publication of this protocol. Some issues, however, should be better clarified in the manuscript, including the following:

Under the Article summary (and Strengths and limitations), they describe their example as one where there are "limited applications". It is not clear if this is saying there are limited observational data sets available for this goal or there are limited opportunities for RCTs, or something else.

Thank you for your comment. We meant that there are limited applications of target trial emulation methodology to estimate treatment effects in CF and so it is a strength that we include an example here. The text has been amended to make this clearer.

Propensity score-based (PS) methods are just one approach for better approximating causal inferences with observational data, augmented inverse PS weighting is just one approach for better balancing confounders, and the logistic model is just one approach for the PS assignment model and for the outcomes model. A better justification should be included for each of these selections. It was also unclear to this reviewer why only confounders are used in the AIPTW calculations (versus including both confounders and outcome predictors to improve precision).

We have included some additional text in the protocol which provides the justifications for our choice to use AIPTW, a doubly-robust approach, rather than outcome-regression methods or propensity score methods (see Section 4.5.3). AIPTW can be used in combination with machine learning methods for estimating the propensity score and/or the outcome models. We plan to explore these options in further work, but for this analysis decided to use parametric models.

The set of confounders (shown in Figure 2) do include what we would consider to be the main predictors of the outcome, for example baseline FEV. We believe there are no important predictors of the outcome that have been omitted from the analysis.

One significant issue for interpretation was the apparent focus on any differences from the target RCT as weaknesses. In multiple places across the paper, they seem to focus on the goal of matching results from the published target trial. In fact, the limitations described in Section 8 seem to focus specifically on reasons for those differences. The Research question and objectives also list replication as a goal.

While this reviewer understands that achieving very similar results as the RCT is reassuring, finding different results may be a significant strength, e.g., through better representation of more diverse or generalizable study populations or through use of larger data sets that are better powered to detect significant relationships. While they do highlight some of these issues, they all seemed to be highlighted under the heading, or in the context of weaknesses not strengths. The conclusions or other sections of the manuscript should better reflect the benefits of target trial emulation beyond simply matching the original target RCT. Part of that benefit can be described through describing potential participant selection bias in the RCT versus the registries.

Thank you for this comment. We have discussed the reasons for potential differences between the target trial and the trial emulations as limitations as they may reduce our chances of replicating the findings from the target trial. The goal in this study is to investigate whether we are able to replicate, according to our defined criteria, the results from a published RCT. This is similar to the goals of target trial emulations in some other clinical areas, e.g. in the RCT DUPLICATE initiative mentioned above. We agree that in some contexts different findings from a study based on observational data could be because the data better reflect the patient population of interest. In this study, the patient population in our emulated trial will be selected to meet the same eligibility criteria as in the RCT. If we find that we are able to replicate the findings of the RCT for this limited population, this will build evidence that the data could be used to obtain reliable estimates of treatment effects in the wider patient population, which would be a key advantage of trial emulation (of hypothetical trials) using registry data. We have added a paragraph at the end of the limitations section to discuss this.

While randomization in an RCT does have clear advantages to giving an unbiased estimate of the causal effect, that estimate may be using a very biased population. While this reviewer understands that the approach in the established protocol cannot be modified now, it would be worth considering ad hoc analyses (where they are labeled as such) which evaluate whether the estimate of treatment effect varies across study populations that were less represented by the RCT. This may be difficult to accomplish however if the inclusion and exclusion criteria are driving those biases in the enrolled RCT population. At the very least, this point should be further described in the conclusions or elsewhere in the paper.

Thank you for your comment. We do plan to conduct analyses to investigate whether the treatment effects may extend to populations less represented RCT as future research, or whether the effect may differ in different patient subgroups. This is discussed briefly in the "Ethics and dissemination" section.

This protocol describes the first step of our research – the benchmarking step – where we see if we can replicate the results of an existing trial, before extending the work to address questions beyond the original trial. As noted above, our hope is that this first step will help build confidence in the results obtained using these data and the target trial emulation approach.

Additional changes made by the authors

In the original submission, we stated that the secondary outcome, prescription of IV antibiotics, will be treated as a binary outcome. We have since changed this to a time-to-event outcome with hazard ratios and rate ratios as the estimands of interest. The new analysis plan for the secondary outcome matches the analysis used in the target trial, which is our goal. Changes are summarised in Section 4.5.4.

We’ve also updated Figure 1 which summarises which visits we will extract our data from for the analysis, and how this differs between the UK and US data. The updated Figure is attached in our revised submission.
